# Advances in the Function and Regulation of Hydrogenase in the Cyanobacterium *Synechocystis* PCC6803

**DOI:** 10.3390/ijms151119938

**Published:** 2014-10-31

**Authors:** Corinne Cassier-Chauvat, Théo Veaudor, Franck Chauvat

**Affiliations:** UMR8221, CEA, CNRS, University of Paris XI, Institute of Biology and Technology Saclay, Laboratory of Biology and Biotechnology of Cyanobacteria, CEA-Saclay, Gif sur Yvette 91 191, France; E-Mails: corinne.cassier-chauvat@cea.fr (C.C.-C.); theo.veaudor@cea.fr (T.V.)

**Keywords:** hydrogen, bioproduction, cyanobacteria, oxidative stress, regulation, cysteine oxidation, sugar stress, electron transport, ferredoxin

## Abstract

In order to use cyanobacteria for the biological production of hydrogen, it is important to thoroughly study the function and the regulation of the hydrogen-production machine in order to better understand its role in the global cell metabolism and identify bottlenecks limiting H_2_ production. Most of the recent advances in our understanding of the bidirectional [Ni-Fe] hydrogenase (Hox) came from investigations performed in the widely-used model cyanobacterium *Synechocystis* PCC6803 where Hox is the sole enzyme capable of combining electrons with protons to produce H_2_ under specific conditions. Recent findings suggested that the Hox enzyme can receive electrons from not only NAD(P)H as usually shown, but also, or even preferentially, from ferredoxin. Furthermore, plasmid-encoded functions and glutathionylation (the formation of a mixed-disulfide between the cysteines residues of a protein and the cysteine residue of glutathione) are proposed as possible new players in the function and regulation of hydrogen production.

## 1. Introduction

Energy is crucial to modern industry. In 2012, 570 ExaJoules (1 EJ = 10^18^ Joules) of energy was consumed worldwide, of which approximately 80% was generated from burning fossil fuels thereby liberating into the atmosphere about 6 gigatons (6 × 10^9^ tons) of the green-house-generating gas CO_2_. Even hydrogen (H_2_), which has a higher energy content than oil (142 MJ/kg for H_2_ *vs.* 44.2 MJ/kg for oil) and burns cleanly, producing only water as its by-product, is not yet a clean biofuel because it is mostly produced from burning oil [1]. Hence, the pollution problem and the fossil fuel shortfall anticipated to occur during the 21st century make it important to develop new energy sources that are plentiful, renewable and environmentally friendly. Sunlight is naturally attractive as it is the most readily available and inexpensive source of energy on Earth. For instance, the annual solar flux received by Earth—approximately 5 YottaJoules (1 YJ = 10^24^ Joules)—is in huge excess of the 570 ExaJoules used by our society. Consequently, there is a growing interest in processes that could couple the solar energy-powered capture of CO_2_ to the production of energies through the use of photosynthetic organisms, such as microalgae [2,3]. Indeed, production by microalgae of renewable biofuels from nature’s most plentiful resources, solar light, water, mineral salts and CO_2_, is of great interest in recycling CO_2_ and saving arable soils, fertilizers, pesticides and fresh waters for crop production. Cyanobacteria (formerly termed blue-green algae) have the potential for that. First, cyanobacteria are the most abundant photosynthetic organisms of our planet. They colonize most ecological niches, fresh and salt waters, terrestrial and extreme environments (pH, temperature) that counter-select most competing organisms, a primary concern of mass cultivation [4]. Second, cyanobacteria convert captured solar energy into biomass at high efficiencies (3%–9%) [2,5] to produce a large part of the atmospheric oxygen and organic assimilates for the food chain [6]. On a global scale, cyanobacteria fix an estimated 25 Giga tons of carbon from CO_2_ per year into energy dense biomass [7]. To perform this huge CO_2_ fixation, cyanobacteria use 0.2%–0.3% of the solar energy, 178,000 terawatts (1 TW = 10^12^ watts), reaching the Earth surface [8]. Thus, the amount of energy passing through cyanobacteria exceeds by more than 25 times the energy demand of our society; roughly 1000-fold the total nuclear energy produced on Earth. Third, as cyanobacteria tolerate high CO_2_ content in gas streams and they can grow in a variety of locations, they can be used as “low-cost” microbial cell factories for the capture and storage of industrial CO_2_ gas near the sites of industrial productions, thereby reducing transportation costs. Fourth, many cyanobacteria as the unicellular model strain *Synechocystis* PCC6803 have a small sequenced genome amenable to genetic manipulations including with versatile plasmid vectors [9,10,11]. A powerful genetics is welcome as natural (wild-type) cyanobacteria are not suitable biofuel producers because some of the required metabolic pathways are partially lacking or need optimization [3,5].

In cyanobacteria, two enzymes can produce hydrogen, the [Ni-Fe] bidirectional hydrogenase (Hox for hydrogenase oxidation) and the nitrogenase. Both enzymes are sensitive to oxygen and do not occur in all strains, but for H_2_ production the [Ni-Fe] hydrogenase, which does not use ATP [12], is energetically favored over the nitrogenase enzyme that consumes 16 ATP per molecules of H_2_ produced [13].

**Figure 1:**
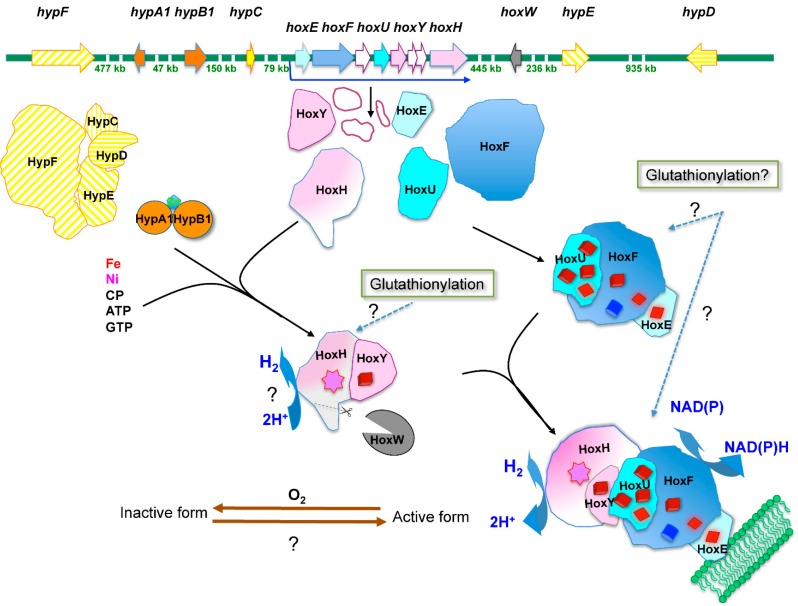
Schematic representation of hydrogen production machine in *Synechocystis* adapted from [12,15]. The genes are represented by arrows, which point in the direction of their transcription, and are colored similarly to their protein products. The green numbers indicate the spacing distance (in kilobases) between the scattered genes. The *hoxEFUHY* operon is weakly transcribed [16] as the polycistronic mRNA (bent blue arrow), which encodes (i) the hydrogenase sub-complex (made by the HoxY protein and the HoxW-matured HoxH subunit); (ii) the HoxEFU diaphorase sub-complex; and (iii) the three proteins of unknown function (white forms). The electron transfer FMN cofactor, Fe-Ni center, and [4Fe-4S] and [2Fe-2S] clusters of the Hox proteins, are represented by the blue squares, the pink star, dark-red squares and light-red diamonds, respectively. The zinc-bound to HypA1 and HypB1 proteins is shown as the blue form. CP designates carbamoyl phosphate. The brown lines stand for the reversible inactivation of Hox activity mediated by oxygen. The photosynthetic membrane is represented in green.

The bidirectional Hox enzyme has been mostly studied in the model cyanobacterium *Synechocystis* PCC6803 (hereafter *Synechocystis*), where it is the sole enzyme capable of combining electrons with protons to produce H_2_ under specific conditions [12,14]. The Hox enzyme comprise five protein-subunits, HoxEFUYH, and a [Ni-Fe] and several [Fe-S] redox clusters (Figure 1). 

The HoxEFU subunits make up the diaphorase sub-complex that transfers to the [Ni-Fe] hydrogenase sub-complex HoxHY the NAD(P)H-transported electrons produced by photosynthesis and/or sugar catabolism [17]. Recently, based on an *in vitro* analysis, it was also proposed that the Hox enzyme can be reduced by ferredoxin or flavodoxin [18], similarly to the [Fe-Fe] hydrogenase of eukaryotic algae [19]. During HoxHY assembly, the HoxH subunit is processed by the HoxW protease [14], and subsequently the [Ni-Fe] HoxEFUYH complex is assembled by the six subunits HypABCDEF complex [12,14]. The Hox enzyme is dispensable to the photoautotrophic growth in standard laboratory conditions [20]; it has a bias towards H_2_ production [21] and it is reversibly inactivated by oxygen [17]. Hence, investigating the photobiological production of H_2_ by cyanobacteria has both an evident biotechnological interest, and a basic research interest, in addressing the paradox of the antagonistic production of hydrogen and oxygen (O_2_ inhibits H_2_ production).

## 2. Expression and Regulation of the Genes Involved in Hydrogen Production

**Figure 2:**
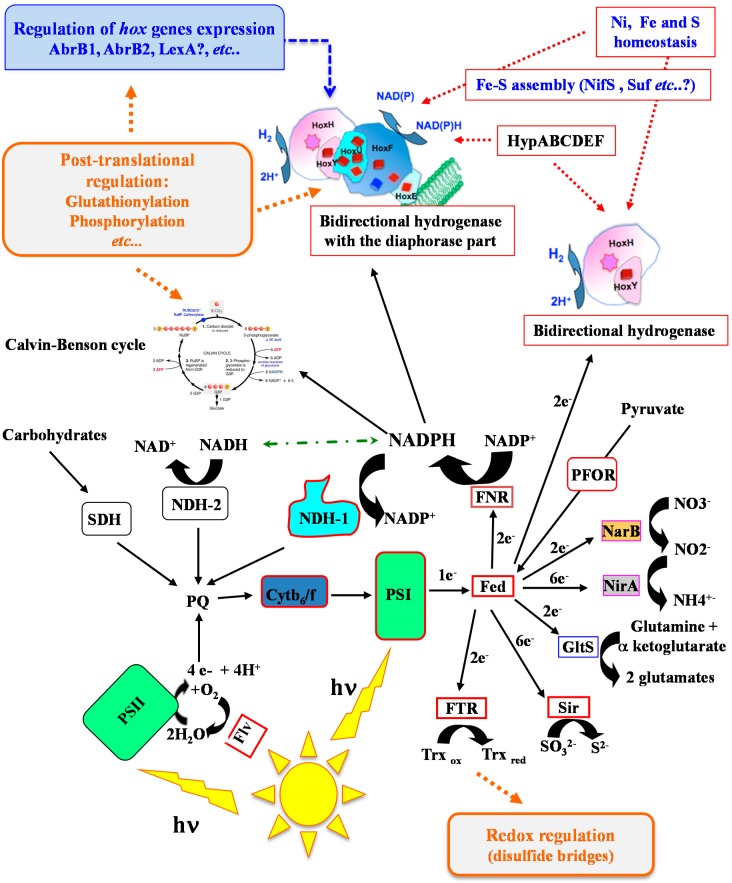
Schematic representation of the redox (electron transfer) metabolism of *Synechocystis* growing in photoautotrophic conditions. The electron transfers and transcriptional regulations are represented by solid black arrows and dotted blue arrows, respectively. The redox dependent- and metal requiring-processes are indicated by orange and red dotted arrows, respectively. PSII, photosystem II; PSI, photosystem I, Cytb_6_/f, cytochrome b6/f; Fed, ferredoxin; Flv, flavodiiron protein; FNR, ferredoxin NADP reductase; FTR, ferredoxin thioredoxin reductase; GltS, ferredoxin dependent glutamate synthase; NarB, Nitrate reductase; NirA, Nitrite reductase; NDH1, NADPH oxidoreductase complex 1; NDH2, NADPH oxidoreductase type 2; PQ, plastoquinone pool; SDH, succinate dehydrogenase; Sir, sulfite reductase; Trx = Thioredoxin.

In order to use cyanobacteria for the biological production of hydrogen, it is important to thoroughly study the regulation of the hydrogen-production machine in order to better understand its role in the global cell metabolism (Figure 2) and identify bottlenecks limiting H_2_ production. 

In contrast to the *hypABCDEF* genes that are scattered in the *Synechocystis* chromosome (Figure 1), the *hoxEFUYH* genes are clustered in an octacistronic operon, which comprises the *hoxE*, *hoxF*, *sll1222*, *hoxU*, *hoxY*, *ssl2420*, *sll1225* and *hoxH* genes in that order, and also encode three proteins of unknown function: Sll1222, Ssl2420 and Sll1225 [14,20]. This operon is weakly expressed as a polycistronic transcript, which initiates 168 bp upstream of the start codon of the *hoxE* gene [22,23]. The promoter of the *hoxEFUYH* operon is not very active [16] though it harbors the two sequences resembling canonical *E.coli*-like promoter boxes, –35 (TTGctc) and –10 (TAacAa) located at correct distances from each other (18 bp) and from the transcription start site (7 bp). As such canonical promoters were long ago shown to be strongly active in *Synechocystis* [24], this indicated that it is negatively regulated (see below).

The *hoxEFUYH* operon is regulated by various environmental conditions, such as hydrogen, light, nitrate, nickel, oxygen and sulfur availabilities [25], in agreement with the finding that H_2_ production can be increased in media with optimized concentrations of these elements [26]. The transcript levels of all *hox* genes are increased (five to sixfold) under microaerobic conditions [27,28], with an additional induction (10–12-fold) of *hoxEF* in darkness [27].

The upregulation of *hox* genes elicited by nitrogen starvation is consistent with the increased H_2_ production triggered by inhibition of nitrate assimilation, suggesting that hydrogenase serves as an alternative sink for photosynthetic electrons no longer used for nitrate reduction [29].

The *hox* genes are also regulated by inhibitors of the photosynthetic electron transport chain in a way suggesting that the redox state of the plastoquinone pool does not play a prominent role in *hox* regulation [27] and H_2_ production [26]. Furthermore, the expression of the *hoxEFUYH* operon is controlled by three transcription factors (Figure 2); two positively acting regulators, LexA (Sll1626) [22,23] and AbrB1 (Sll0359) [30], and one repressor AbrB2 (Sll0822) [16].

### 2.1. LexA a Positively Acting Player in Hydrogen Production

By using streptavidin-coated magnetic beads in DNA affinity assays two groups identified the LexA protein as capable of binding to two regions of the *hox* promoter region, *i.e.*, at 198–338 bp [23] or 592–690 bp [22] upstream of the *hoxE* start codon. Prior to these studies, the LexA regulator was found not to regulate typical DNA repair genes, unlike what occurs in *E. coli*, but instead to control genes involved in carbon metabolism [31] and the redox-responsive gene *crhR* encoding an RNA helicase involved in translational regulation [32]. Furthermore, proteomic analyses identified LexA as protein associated to plasma membrane and thylakoids [25].

The occurrence of two LexA binding sites in the *hox* promoter suggest that the LexA-mediated regulation involves DNA bending [14], a well-known phenomenon in transcriptional regulation. Furthermore, the depletion of the LexA protein decreased the hydrogenase activity, suggesting that LexA plays a positive role in hydrogen production [25]. To test the influence of LexA on the transcription of the *hox* operon, we cloned the *hox* promoter in front of the promoter-less *cat* reporter gene of the promoter-probe plasmid vector pSB2A, which replicates stably in *Synechocystis* at one copy per chromosome copy [9]. Then, we introduced the corresponding recombinant plasmid pSB2A-*hox* promoter in the WT (wild-type) strain and Δ*lexA* mutants where we measured the expression of the *cat* (chloramphenicol acetyl transferase) gene driven by the *hox* promoter. Similar CAT activities were obtained in the WT and Δ*lexA* strains growing in standard photoautotrophic conditions, indicating that the activity of the *hox* promoter is not affected by the absence of LexA (data not shown). This finding challenges the notion that LexA is a direct transcription regulator of the *hox* operon.

### 2.2. The AbrB1 Protein Positively Regulates the Transcription of the hoxEFUHY Operon

By using DNA affinity assays, another transcription factor positively acting on *hox* expression was isolated on the basis of its specific interaction with the *hox* promoter region. This regulator, AbrB1, resembles the AbrB regulator of *B. subtilis*, which regulates about 100 genes involved in various processes (sporulation, biofilm formation, antibiotic production, development of competence for DNA uptake), though the cyanobacterial AbrB1 protein has its putative DNA-binding domain in its *C*-terminal region, instead of in the usual *N*-terminal region as occurs in non-cyanobacterial AbrB proteins [30,33]. The *Synechocystis* AbrB1 protein was found to be indispensable to cell life in both the wild-type strain [30] and the glucose tolerant mutant [33]. Interestingly, AbrB1 was found to interact with a high degree of confidence with itself, suggesting that AbrB1 acts as an oligomer [25]. The *abrB1* gene is expressed from an atypical promoter harboring an extended –10 element (5'-tgtTATAtT-3') [30], which might compensate the absence of a genuine –35 box (5'-TTGACA-3'; located at the correct 17 ± 1 bp distance from the –10 element) similarly to what found for the *secA* gene [34].

### 2.3. The AbrB2 Repressor of the hoxEFUYH Operon Plays a Prominent Role in the Regulation and the Tolerance to Oxidative and Metal Stresses

Because it resembles to AbrB1, the AbrB2 has also been studied in *Synechocystis*, and appeared to be dispensable to the growth of both the glucose-tolerant mutant [33] and the wild-type strain [16]. In the glucose-tolerant mutant, AbrB2 appeared to regulate numerous genes involved in nitrogen and carbon assimilation, and to interact with AbrB1 [33,35,36]. In the wild-type strain AbrB2 was shown to repress its own gene and the *hox* operon [16]. The others genes directly involved in hydrogen production: *hoxW*, *hyp*, *lexA* and *abrB1* were not regulated by AbrB2, even though it appeared to regulate a large number of genes (about 330 genes, mostly negatively), as shown by transcriptome analysis with pan-genomic microarrays [37]. The highest number of the AbrB2-responsive genes code for hypothetical proteins or unknown proteins (about 200 genes), emphasizing that we still have a limited knowledge of *Synechocystis*, though it is one of the more intensively studied cyanobacterium. Many these unknown genes belong to the endogenous plasmids, suggesting that they play a role in hydrogen production. 

The AbrB2 master regulator was also found to regulate several transport genes, including *cysAPUW* (SO_4_^2−^), *fecBCD* (Fe^2+^), in agreement with the Hox enzyme using a Ni-Fe and several Fe-S redox clusters to produce hydrogen [37]. In this *abrB2*-deleted mutant, the increased expression of the anti-oxidant genes *cydAB* (cytochrome bd-quinol oxidase that can reduce O_2_) and *norB* (nitric oxide reductase) are consistent with the increased production of hydrogen [16], which is regarded as an anti-oxidant process that evacuates electrons occurring in excess to prevent them to reduce molecular oxygen and produce toxic reactive oxygen species [12,15,17,18]. Consistently, the absence of the AbrB2 repressor led to an increased tolerance of *Synechocystis* to both metal and oxidative stresses triggered by nickel and the thiol oxidizing agent diamide [38]. 

The *abrB2* promoter region was also analyzed, using transcriptional fusion to the promoter-less *cat* reporter gene of the plasmid pSB2A that replicates stably in *Synechocystis* at one copy per chromosome copy [9]. The *abrB2* gene was found to expressed from an atypical promoter, which harbors an extended –10 element (5'-TGTATAAT-3'), likely compensating the absence of a genuine –35 box (5'-TTGACA-3'; located at about 17 ± 1 bp from the –10 element), similarly to the genes *secA* [34], *gap2* [39] and *abrB1* [30]. In addition, a consensus DNA motif, TT-(N_5_)-AAC, was also proposed for AbrB2 binding [16].

## 3. Function and Over-Production of the Hydrogen Production Machine

To attempt increasing hydrogenase activity in *Synechocystis*, Germer and co-workers used the light-inducible promoter of the photosynthetic gene *psbAII* to increase the expression of the endogenous *hoxEFUYH* operon and the heterologous _Nos_*hypABCDEF* operon from *Nostoc* PCC7120 [40]. The gain in activity was modest (3.2-fold; *i.e.*, from 2.9 nmol H_2_·min^−1^·mg·chlorophyll^−1^ in wild-type cells up to 9.4 nmol H_2_·min^−1^·mg·chlorophyll^−1^ in mutant cells) for three main possible reasons. First, the light-inducible *psbAII* promoter used to increase the expression of the *hoxEFUYH* and the _Nos_*hypABCDEF* genes is more active under high light that increases the photosynthetic production of O_2_, which inhibits hydrogenase activity. Second, the *Nostoc* HypABCDEF proteins might be not fully active on the *Synechocystis* PCC6803 HoxEFUYH proteins. Third, it is also possible that the *Nostoc* HypABCDEF proteins might somehow interfere with the function of the endogenous *Synechocystis* HypABCDEF proteins.

To increase the expression of the *hoxEFUYH* operon and the *hypABCDEF* genes, we have used the strong λ*p_R_* promoter [10,24]. Practically, we replaced the weak [16] natural promoter of the *hoxEFUYH* operon by the λ*p_R_* promoter, and we cloned the *hypABCDEF* genes under the control of the same λ*p_R_* promoter, in a plasmid that replicates at the same 10–20 copies per cell as the chromosome [10]. The mutant grew as fit as the WT strain. It strongly over-expressed the *hoxEFUYH* operon and the *hypABCDEF* genes (about 100-fold), and exhibited a 20-fold higher levels of active hydrogenase than the WT strain. These findings show that an increased production of active hydrogenase is not detrimental to cell life, and other factors than *hoxEFUYH* and *hypABCDEF* transcription limit hydrogen production (see below).

## 4. Role of the Hydrogen Production Machine

Though, the complex hydrogenase enzyme is neither ubiquitous in all cyanobacteria nor essential to the standard photoautotrophic growth of model strains [12], the analysis of gene deletion and overexpression mutants showed that the presence of the Hox enzyme offers some growth advantages in certain environmental situations.

### The hoxEFUYH Operon Operates in the Protection against Redox Stresses Triggered by Hydrogen Peroxide or the Reduced Carbon Metabolites Glucose and Glycerol

All aerobic organisms invariably produce reactive oxygen species, such as H_2_O_2_, through the accidental autoxidation of redox enzymes [41], which occurs when their reduced cofactors reduce oxygen. This phenomenon is important in cyanobacteria, because their active photosynthesis massively produces oxygen and electrons [42]. As the cyanobacterial hydrogenase enzyme complex has been proposed to act as an electron valve releasing some of the supernumerary electrons [12,17], we have compared the H_2_O_2_ tolerance of the WT strain to that of mutants which either lack the *hoxEFUYH* operon, or overexpress it, alone or in combination with the *hypABCDEF* genes [15]. The data showed that the *hoxEFUYH* operon and the *hypABCDEF* genes contribute to the protection against H_2_O_2_, positively (*hoxEFUYH* operon) or negatively (*hypABCDEF*). Future experiments will be required to test whether the higher H_2_O_2_ tolerance directed by the overexpression of the *hoxEFUYH* operon is due to the increased abundance of (i) the HoxHY hydrogenase enzyme *per se*; (ii) the HoxEFU diaphorase enzyme; and/or (iii) the Sll1222, Ssl2420 and Sll1225 proteins of as yet unknown function.

In addition, we have tested the influence on hydrogenase deletion or overproduction mutants of the reduced-carbon metabolites glucose and glycerol, a cheap surplus of industries [43] that stimulate hydrogen production in the cyanobacteria *Arthrospira* (*Spirulina*) *maxima* [44] and *Cyanothece* ATCC 51142 [45]. Both glucose and glycerol might be toxic to *Synechocystis* growing under an otherwise normal light fluence, probably because these reduced metabolites somehow decrease the electrons-consuming CO_2_-assimilation, thereby allowing spared electrons to recombine with O_2_ and generate ROS [46]. The level of tolerance to these stresses increased in parallel with the abundance of the Hox proteins [15], in agreement with the higher tolerance to a mixture of both glucose and arginine of the WT strain as compared to a *hoxH*-deletion mutant [18]. Collectively, these data are consistent with the proposal that hydrogenase operates as an electron valve preventing supernumerary electrons generated by photosynthesis and/or sugar catabolism to recombine with O_2_ to generate toxic reactive oxygen species [12].

## 5. Glutathionylation a Probable New Player in Hydrogen Production

As mentioned above, cyanobacteria are continuously challenged with toxic reactive oxygen species generated by photosynthesis and respiration, which can oxidize the thiol group (SH) of two cysteinyl residues to form disulfide bonds (-S-S-) between proteins, or between a protein and a molecule of the, crucial [47], anti-oxidant tripeptide glutathione (glutathione-protein mixed disulfide, also termed glutathionylation) [41,42]. Collectively, the data suggesting that the *hox* operon repressor AbrB2 exists under two uncharacterized posttranslational modification forms [35,48], and the findings that AbrB2 and the *hox* operon play a role in the tolerance to oxidative stresses [15,38], prompted us to test whether these proteins might be glutathionylated. We found that the single, widely conserved cysteine of AbrB2 is the target of glutathionylation, which affects the oligomerization of AbrB2; its binding on the *hox* operon-promoter DNA; its repression of a wealth of other genes; and its stability at the non-standard temperature of 39 °C [38]. The role of AbrB2 can be viewed as follows. In cells growing in absence of stress, AbrB2 down-regulates hydrogen production and other stress defenses. By contrast, in cells facing oxidative stresses triggered by light excess or metal availabilities, AbrB2 is oxidized and its single cysteine is glutathionylated. Thus, AbrB2 is no longer able to repress hydrogen production thereby allowing evacuation of excess electrons, among other anti-oxidant processes. After recovery from oxidative stress, AbrB2 activity is restored (likely through deglutathionylation catalyzed by glutaredoxins [49]) and starts repressing hydrogen production and other anti-oxidant process, to save photosynthetic electrons. Similarly, we found that the cysteine of the other *hoxEFUYH* regulator, AbrB1, which interacts with AbrB2 and also regulates the *hoxEFUYH* operon [30,33,36], can be glutathionylated *in vitro* like AbrB2. Thus, it will be interesting in the future to analyze the influence of the glutathionylation of AbrB1 and AbrB2 on their interaction and their regulation of hydrogen production. To our knowledge, AbrB2 and AbrB1 are the first cyanobacterial regulators reported to undergo glutathionylation (in other prokaryotes, only the OxyR regulator is known to be controlled by glutathionylation [50]), emphasizing on the evolutionary conservation of this process, well described in eukaryotes [51].

Stimulated by the findings that both AbrB1 and AbrB2, and the mercuric reductase enzyme [49], can be glutathionylated, we performed a large-scale proteomic analysis of glutathionylation [52]. Amongst the glutathionylatable proteins, we found HoxH and HoxF. Interestingly, the glutathionylated Cys (C289) of HoxF is located close to the putative binding site for NAD(P). These findings will certainly stimulate the analysis of the crosstalk between hydrogen production and the oxidative stress-responsive glutathionylation-and-deglutathionylation process. 

## 6. Conclusions

Though significant progress has been made recently in our understanding of the function and regulation of the complex hydrogen Ni-Fe hydrogenase of the model cyanobacterium *Synechocystis* PCC6803, important questions remain.

Recent *in vitro* data suggested that the Hox enzyme can receive electrons from not only NAD(P)H as usually shown, but also, or even preferentially, from ferredoxin. This proposal will certainly stimulate the investigation of the selectivity/redundancy of the nine ferredoxins of *Synechocystis*, which are conserved in cyanobacteria.

Using gene deletion and over-expression, it has been shown recently that the *hoxEFUYH* operon operates in the defense against redox stresses triggered by H_2_O_2_ or reduced carbon or nitrogen metabolites. These findings strengthen the proposal that hydrogenase operates as an electron valve to prevent the supernumerary electrons to recombine with O_2_ to generate toxic reactive oxygen species. Hence, the hydrogenase complex can be viewed as an important enzyme in cyanobacteria like *Synechocystis*, which can grow in biofilm, a thick network of auto-aggregated cells where they are inevitably exposed to H_2_O_2_ and reduced metabolites released by their neighbors (living or dying and lysing). This view is strengthened by the absence of hydrogenase enzyme in most planktonic cyanobacteria living in open oceans. It will be interesting in the future to investigate the influence of the *Synechocystis* hydrogenase enzyme in the growth and response to environmental stresses of cells incubated not only in agitated liquid cultures but also in static conditions or on solid media to favor the formation of biofilm.

The recently achieved simultaneous overproduction of the HoxEFUYH and HypABCDEF proteins within the same cells led to a 20-fold increase in active hydrogenase. These sophisticated mutants with a higher hydrogenase content and a healthy growth will be very useful cell factories for the purification of large hydrogenase quantities for structural analyses, the data of which should enable the design of a meaningful mutational strategy to increase the low natural O_2_ tolerance of the hydrogenase enzyme to increase hydrogen production. 

All hydrogenase-overproducing mutants displayed higher levels of expression of the *hoxEFUYH* and *hypABCDEF* genes than that of active hydrogenase, indicating that limiting post-transcriptional factors should be dealt with. Thus, it will be important to pay particular attention to glutathionylation (the formation of a mixed-disulfide between the cysteines residues of a protein and the cysteine residue of glutathione) because it was recently reported that the AbrB1 and AbrB2 hydrogen regulator and the HoxH (diaphorase) and HoxF (hydrogenase) protein-subunits can be glutathionylated. These findings will certainly stimulate the analysis of the redox crosstalk between hydrogen production and the oxidative stress-responsive glutathionylation-and-deglutathionylation process. 
